# The Role of DNMT Methyltransferases and TET Dioxygenases in the Maintenance of the DNA Methylation Level

**DOI:** 10.3390/biom14091117

**Published:** 2024-09-04

**Authors:** Anastasiia T. Davletgildeeva, Nikita A. Kuznetsov

**Affiliations:** 1 Institute of Chemical Biology and Fundamental Medicine, Siberian Branch of Russian Academy of Sciences, 630090 Novosibirsk, Russia; nikita.kuznetsov@niboch.nsc.ru; 2 Department of Natural Sciences, Novosibirsk State University, 630090 Novosibirsk, Russia

**Keywords:** DNA demethylation, epigenetics, methylome, DNA dioxygenase, DNA methyltransferase, catalytic mechanism, single-nucleotide polymorphism, enzyme disfunction

## Abstract

This review deals with the functional characteristics and biological roles of enzymes participating in DNA methylation and demethylation as key factors in epigenetic regulation of gene expression. The set of enzymes that carry out such processes in human cells is limited to representatives of two families, namely DNMT (DNA methyltransferases) and TET (DNA dioxygenases). The review presents detailed information known today about each functionally important member of these families and describes the catalytic activity and roles in the mammalian body while also providing examples of dysregulation of the expression and/or activity of these enzymes in conjunction with the development of some human disorders, including cancers, neurodegenerative diseases, and developmental pathologies. By combining the up-to-date information on the dysfunction of various enzymes that control the DNA “methylome” in the human body, we hope not only to draw attention to the importance of the maintenance of a required DNA methylation level (ensuring epigenetic regulation of gene expression and normal functioning of the entire body) but also to help identify new targets for directed control over the activity of the enzymes that implement the balance between processes of DNA methylation and demethylation.

## 1. Enzymatic Systems That Ensure the Balance of Epigenetic Methylation and Demethylation of DNA

One of the most common forms of epigenetic modifications in living organisms is methylation [1]. DNA methylation at the C5 position of cytosine in the context of CpG dinucleotides underlies the regulation of many biological processes, including the expression of gene products, X chromosome inactivation, and a number of other vital phenomena in mammalian cells [2]. In general, epigenetic DNA methylation leads to transcriptional repression of genes through a number of mechanisms, such as transcription factor binding [3], recruitment of specialized proteins binding methylated DNA, and chromatin-modifying proteins [4]. The maintenance of proper levels of DNA methylation is crucial for normal (healthy) development and for homeostasis in mammalian species. The maintenance of an optimal level of DNA methylation requires coordinated actions of a system of enzymes that introduce methyl groups (DNA methyltransferases) and remove these groups (demethylases) and various proteins that associate with methylated DNA [1]. DNA methyltransferases 3 (DNMT3A and DNMT3B) can attach the methyl group to atom C5 of cytosine in CpG dinucleotides de novo, while DNMT1 has a preference for hemimethylated DNA strands and upholds the corresponding pattern of DNA methylation on a freshly synthesized strand after replication. Active demethylation of m^5^C proceeds through enzymatic modification of the methyl group of m^5^C, and this reaction is implemented by representatives of the dioxygenase family—namely ten-eleven translocases (TET1/2/3)—followed by processing of m^5^C oxidation products through the BER (base excision repair) pathway [5,6,7]. TET enzymes are affiliated with a superfamily known as “Fe(II)/α-ketoglutarate (αKG)-dependent dioxygenases” and catalyze the conversion of m^5^C into 5-hydroxymethylcytosine (hm^5^C) [7,8] and then into 5-formylcytosine (f^5^C) and 5-carboxylcytosine (ca^5^C), which are removed from DNA via BER to restore the original sequence [8,9,10]. It should be noted that in the current review, we focus mainly on mammalian enzymes from the DNMT and TET families.

## 2. DNA Methyltransferases

Enzymatic methylation of cytosine in CpG dinucleotides is performed by DNA methyltransferases, which catalyze the addition of a methyl group at the C5 of cytosine. In humans, three canonical isoforms have been identified, namely DNMT3A, DNMT3B, and DNMT1. It has also been shown that DNMT3A is expressed as two splice isoforms: DNMT3A1 and DNMT3A2. The main structural difference is that DNMT3A2 is shorter by 219 N-terminal amino acid residues [11]. DNMT2 and DNMT3L are noncanonical members of the family because they do not possess the catalytic activity of DNMTs [12]. DNMT1 plays an important part in the maintenance of an existing DNA methylation pattern, whereas DNMT3A and DNMT3B carry out de novo DNA methylation [12,13]. Nonetheless, some studies have shown that DNMT3B and DNMT3A can also implement the preservation of a current DNA methylation level [14,15]. Moreover, methyltransferase DNMT3C was discovered in male germ cells of rodents and protects these cells from transposon activity. The *Dnmt3c* gene has arisen in the rodent genome owing to duplication of the gene encoding DNMT3B [16].

**Figure 1:**
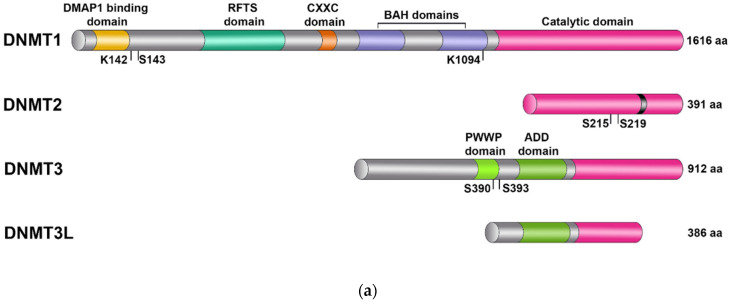

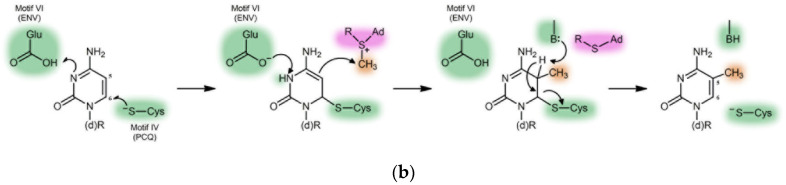
(**a**) Conserved domains in members of the mammalian DNA methyltransferase family. All DNMT enzymes have a conserved catalytic domain (pink). A minor subdomain inside DNMT2’s catalytic domain (black) represents a one-of-a-kind CFT motif that is characteristic of only this member of the DNMT family. The numbers of amino acid residues presented for every protein correspond to human homologs, whereas DNMT3A is shown as an example of DNMT3 enzymes. The amino acid residues indicated below the structures represent positions of documented post-translational modifications. (**b**) The mechanism of catalytic action of DNMT enzymes. The methylation reaction is initiated when a conserved cysteine residue of motif IV (i.e., the PC motif) of a DNMT enzyme takes part in a nucleophilic attack on atom C6 of the cytosine ring; this attack is also promoted by a conserved glutamate residue within motif VI (i.e., the ENV motif). Following the attack, there is the transfer of the methyl from *S*-adenosyl methionine (SAM) to atom C5 of the cytosine ring and deprotonation of C5 followed by m^5^C formation, which is thought to be mediated by a basic group (presented as “B”:) provided by the enzyme. The amino acid residues participating in the reaction are green, SAM is marked in violet, and the methyl group is orange. BAH: bromo-adjacent homology; ADD: ATRX–DNMT3–DNMT3L; RFTS, replication foci-targeting sequence; and DMAP1: DNA methyltransferase 1-associated protein 1.

The structure of DNMT enzymes usually includes a regulatory N-terminal domain and a catalytic domain at the C terminus (Figure 1). The exception is DNMT2, which is composed exclusively of the catalytic domain. The C-terminal catalytic domain contains 10 small sequence motifs, and six of them (I, IV, VI, VIII, IX, and X) are highly conserved among all DNA methyltransferases [17]. The N-terminal part of DNMT1 has a few conserved smaller subdomains that participate in molecular interactions, including binding to transcription proteins, to a replication locus, and to unmethylated DNA [12]. Conserved domains have also been identified in the structure of DNMT3. These domains are responsible for the interaction of this protein with chromatin [12].

All catalytically active DNMT enzymes use *S*-adenosyl methionine (SAM) as a donor of a methyl group. DNMTs implement the catalysis by everting a target base into the active site of the enzyme [12]. The latest hypothesized mechanism of the DNA methylation reaction is presented in Figure 1b [18]. It should be noted that the one-carbon metabolism in which SAM is produced should significantly alter DNA methylation homeostasis. It is known that SAM is the sole methyl group donor involved in the methylation of DNA, RNA, and proteins [19]. SAM is involved not only in transmethylation but also in *trans*-sulfuration, polyamine synthesis, and 5′-deoxyadenosyl 5′-radical-mediated biochemical transformations. Moreover, the reaction scope can be expanded because SAM itself can be modified prior to the transfer of the chemical group [20]. An abnormal methyl cycle and related methylation processes can be caused by genetic alterations, toxic chemicals, or dietary deficiencies and contribute to the etiology of many pathologies including cancer [21,22], diabetes mellitus [23], atherosclerosis and cardiovascular diseases [24,25], birth defects [26], and neurodegenerative diseases [27]. Because the SAM level is a key regulator of important biochemical pathways, modulation of SAM concentration is also considered a new therapeutic strategy for some diseases [19,28,29].

Given that the activity of DMNTs is extremely important for the whole organism, it is subjected to control by multiple mechanisms. For example, although DNMT3L is an inactive copy of DNMT3, DNMT3L acts as a regulator of DNMT3A activity via the formation of a heterotetramer consisting of two molecules of DNMT3L and a pair of DNMT3A molecules. Such a heterotetramer with DMNT3A at the center has a stronger affinity for DNA, and this property makes DNA methylation more efficient [12,30]. It has also been shown that DNMT3L activates DNMT3B through direct interaction with the enzyme [31]. The activity of DNMT enzymes is also regulated through post-translational modification and alternative splicing [12].

Another interesting example of variations of DNMTs’ activity in various organisms involves changes in the copy number of genes encoding these enzymes. Due to the large number of deciphered insect genomes, it is possible to examine the evolution of *Dnmt* genes in this class of animals [32]; for example, analysis has uncovered an increase in the copy number of *Dnmt1* in *Apis mellifera* (Western honey bee), inactivation of *Dnmt3* in the domestic silk moth *Bombyx mori*, and inactivation of both *Dnmt3* and *Dnmt1* in *Drosophila melanogaster*. Of note, the genome of some laboratory animals, in particular of the popular experimental model *Caenorhabditis elegans* (roundworm), has completely lost the *Dnmt* family in the course of evolution [12,32].

One of the possible factors influencing enzymatic activity is single-nucleotide polymorphisms (SNPs) of the genes. It is known that SNPs represent the most common type of genetic variation in humans [33]. Experimental data on some polymorphic variants (of DNMT enzymes) associated with diseases are summarized in Table 1 and are examined in more detail in subsections below.

### 2.1. DNMT1

#### 2.1.1. Functional Characteristics

DNMT1 is the only methyltransferase known to maintain already established DNA methylation patterns. This enzyme consists of 1620 amino acid residues, which constitute an extended N-terminal regulatory domain and a more compact catalytic C-terminal domain [12]. The N-terminal domain of DNMT1 contains a subdomain binding to DNA methyltransferase-associated protein 1 (DMAP1), cysteine-rich subdomain CXXC, RFTS (replication foci-targeting sequence), and a Bromo homology domain (BHD). The DMAP1-binding domain mediates transcriptional co-repression through interaction with HDAC2 [49]. The RFTS domain is involved in DNMT1 dimerization [50], regulates the binding of DNMT1 to hemimethylated DNA during the S phase of the cell cycle, and is required for its continued binding to heterochromatin in the G2 phase. DNMT1 has a preference for unmethylated CpG dinucleotides on a newly synthetized DNA strand, whereas the RFTS domain serves as a controller by precisely directing DNMT1 to hemimethylated sites. At the same time, the CXXC domain ensures the interaction of DNMT1 with unmethylated DNA [51]. The DNA methyltransferase activity is provided by the C-terminal catalytic domain. Allosteric regulation of the DNA methyltransferase function of DNMT1 is thought to require a proper interaction between the N-terminal regulatory domain and the catalytic C-terminal domain of the enzyme [52,53].

#### 2.1.2. The Role in the Organism

Methyltransferase DNMT1 is absolutely necessary for normal embryonic development and has critical functions in the maintenance of chromatin structure, neuronal survival, and cell cycle regulation [54]. During embryogenesis, the maternal genome largely loses DNA methylation through a replication-dependent phenomenon involving the exclusion of DNMT1 (maintenance DNA methyltransferase) from the nucleus [55]. After cell specification is complete in the developing embryo, the finished DNA methylation patterns established by DNMT3 are maintained by methyltransferase DNMT1. This phenomenon relies on the selectivity of DNMT1 for hemimethylated DNA strands with CpG dinucleotides [56,57]. On the other hand, DNMT1 poorly recognizes hm^5^C, f^5^C, and ca^5^C, and for this reason, the oxidation of m^5^C can lead to the loss of methyl marks through further passive demethylation linked with replication [58].

#### 2.1.3. Single-Nucleotide Polymorphisms (SNPs), Deletions, and Suppression of Activity

With rare exceptions, aging in mammals is often associated with hypomethylation of CpG dinucleotides, especially in regions of DNA repeats. This phenomenon appears to be at least partially involved in the diminution of heterochromatin during aging. The methylation loss within DNA CpG-rich regions in repeated sequences increases the risk of activation of dormant retrotransposons, thereby leading to genomic instability during aging because retrotransposons constitute the lion’s share of DNA repeats [59]. The overall decrease in DNA methylation levels during aging may be due to diminished levels of maintenance methyltransferase DNMT1 in the cell [60]. A recent analysis also revealed a DNA methylation loss in *cis*-regulatory elements, e.g., enhancer regions within β cells (pancreas), and suggests that binding of transcription factors may interfere with the capacity of DNA methyltransferases eventually to recognize these sites, thereby leading to abnormal expression of genes in aged pancreatic cells [61].

Research in recent decades has shown that in cancer and various developmental disorders, diverse changes in genes *Dnmt*, *Tet*, and *Mecp2* (the gene coding for a protein that associates with methylated DNA) are often detected [62]. Mutations in the *Dnmt1* gene result in a significant decrease in the amount of m^5^C in embryonic stem cells, while no visible abnormalities of cell growth rates or morphology are detectable [63]. Nevertheless, *Dnmt1* deficiency in mice is embryonically lethal, and such embryos die in mid-gestation, thus pointing to the important participation of DNMT1 in embryonic early development, which is concurrent with epigenetic reprogramming.

Fibroblasts featuring low amounts of DNMT1 have been found to lose more than 95% of all methyl marks on DNA, likely because the insufficient maintenance activity cannot replace all the methyl marks lost during replication [64]. Notably, however, in these cells, the promoter of gene *Oct3/4* is still methylated; this is probably because its affinity to de novo methyltransferases can compensate for the DNA methylation loss that occurs normally during DNA replication and cell proliferation [65]. In fact, it has been mostly proven that de novo methyltransferases may partially compensate for the reduced activity of DNMT1 in DNA methylation maintenance [66,67].

The functions of methyltransferases can be impacted by diverse factors. For instance, in systemic lupus erythematosus, aberrations of DNA methylation are associated with DNMT1 expression, while significantly lower amounts of DNMT1 and DNMT3A transcripts are observed. In diseases that cause under-expression or hypofunction of DNMT1, the DNA methylation pattern is not completely transferred to a newly created cell after mitosis, resulting in passive demethylation [68].

In the *DNMT1* gene, 11 nonsynonymous mutations have been discovered whose presence is characteristic of patients with disorders of the central and peripheral nervous systems. Moreover, all these mutations are localized to two exons (20 and 21), which code for DNMT1’s domain responsible for binding to chromatin [34,35,36,37,39].

For instance, in exon 21 of the *DNMT1* gene, de novo polymorphisms have been identified that underlie the deafness, narcolepsy, and ataxia that are seen in autosomal dominant cerebellar ataxia, deafness, and narcolepsy (ADCA-DN) [38]. All three polymorphic variants produce amino acid substitutions A570V, V606F, or G605A, which are located in the RFTS domain, affect the protein’s folding, and weaken the interaction between the RFTS domain and the catalytic domain, thereby causing reduced enzymatic activity, insufficient methylation of DNA CpG sites, and incomplete gene silencing.

The *DNMT1* mutations leading to substitution Y495C or double substitution D490E/P491Y have been found in patients with hereditary sensory autonomic neuropathy type 1E (HSAN1E) [35], which features autonomic and sensory neuropathy along with dementia and hearing loss. Additionally, in 2015, Baets and colleagues [54] detected seven different missense mutations of DNMT1 (C353F, P491L, T481P, Y524D, Y495C, I531N, and Y495H) in patients with HSAN1E, five of which were found for the first time (T481P, C353F, Y524D, P491L, and I531N). All seven substitutions are located in the regulatory portion of the RFTS of the N-terminal domain, and all were predicted to be “deleterious” according to SIFT (score: 0), “likely deleterious” according to PolyPhen-2 (P = 1), and “disease-causing” according to MutationTaster (P = 1). Those authors attribute the development of HSAN1E in the presence of DNMT1 mutations not only to a disturbance of the general level of DNA methylation but also to the formation of incorrectly folded mutants of the DMNT1 protein, which provoke aggresome-induced autophagy [54].

Aside from disorders of peripheral and central nervous systems, DNMT gene polymorphisms have been found to correlate with the risk of breast cancer [69]. For instance, SNP rs16999593, which produces missense substitution H97R in DNMT1, is associated with a high risk of breast cancer [40]. In general, however, reports of DNMT genes’ mutations being associated with various diseases are quite rare, consistently with their overall importance and versatile activities and suggesting strong selection against loss-of-function mutations.

An increased expression level of DNMT1 has been found in tissue samples from metastatic breast tumors, whereas upregulation of DNMT3A and DNMT3B has been observed mainly at an early stage of tumorigenesis, implying a tumor stage-dependent expression pattern of these enzymes [70]. Nonetheless, it is known that aside from de novo DNA methylation, tumorous cells show a high degree of demethylation. First reports have created the impression that the reduction in DNA methylation levels in tumor cells is global and hence may include the majority of the non-CpG-island loci in the genome that are typically methylated in each cell type [71]. Nevertheless, recent studies more precisely determined the topography of this demethylation process, and it currently seems that it primarily affects genomic regions that are bound to the nuclear lamina [72,73]. These regions of the genome show late replication and stronger gene repression. Even though the mechanism behind this demethylation is unknown, those authors hypothesized that it may take place as a passive process in which maintenance DNA methylation cannot keep up with rapid replication. Because all free DNMT proteins within a mammalian cell seem to be able to combine into a multipurpose enzymatic complex, it is believed that in tumors, most of DNMT1 may be abnormally recruited to the CpG islands that will be de novo methylated, and this arrangement, in turn, may diminish the magnitude of maintenance activity of DNMT1 in a replication fork, thereby causing passive demethylation [74]. This phenomenon should most strongly affect genomic regions bound to nuclear lamins because only these regions undergo replication late in the S phase. If this mechanism is correct, then it may comprehensively explain why tumorous cells show targeted de novo modification of DNA along with general demethylation [75].

The involvement of enzymes of the DNMT family in human disorders is discussed usually in the context of cancers because alterations of DNA methylation patterns represent some of the earliest molecular alterations in human tumor cells [76,77]. Furthermore, 2′-deoxy-azacytidine and 5-azacytidine are DNMT inhibitors that have already been approved as a therapy for hematopoietic cancers [78]. The clinical efficacy of these therapeutics is impressive already, and their wider use against solid tumors and in combination with immunotherapy may enhance their clinical impact [79,80]. Nonetheless, the mechanisms of action of these inhibitors require further investigation [12].

### 2.2. DNMT3A/3B

#### 2.2.1. Functional Characteristics

After the removal of virtually all methyl marks in the embryonic genome at the early stages of development, a new pattern of DNA methylation becomes established during the implantation of the embryo. This process is mainly mediated by the upregulation of DNMT3A and DNMT3B (de novo DNA methyltransferases) as well as of DNMT1 [13,81]. At the same time, DNMT3 enzymes are not selective, and therefore which regions will be methylated is determined by additional factors, such as the protection of promoter CpG-rich regions and enhancers by bound transcription factors, methylation of histone H3 on lysine 4 (H3K4 methylation), and some proteins [12].

The existence of non-CpG methylation in mammalian DNA is known too [2,82], and it has been revealed that DNMT3A is able to induce methylation at non-CpG sites in embryonic stem cells but not somatic tissues [83]. Because de novo methylation of a DNA molecule is not specific to CpG dinucleotides, it has been theorized that CpG-unrelated methylation has its own epigenetic function; this is because methylation of non-CpG sites is also capable of recruiting methyl-CpG-binding protein 2 (MECP2), i.e., a reader, which is an important transcriptional repressor, in particular the repressor linked with neuronal development [84,85,86,87]. Indeed, human non-CpG methylation patterns show both tissue-specific and inter-individual differences [88,89]. Moreover, it has been suggested that the emergence of non-CpG methylation may have fostered the evolution of sophisticated cognitive abilities found in the vertebrate lineage [90]. Although the biological function of this type of methylation is not fully elucidated and is still debated, at present, high-throughput analyses and improved enrichment methods allow for determining the role of genome-wide non-CpG methylation and reveal its importance [91,92]. The loss of methyl marks in primordial germ cells of the embryo proceeds through passive demethylation due to significant transcriptional suppression of the genes of DNMT3A and DNMT3B (de novo methyltransferases). Moreover, the role of the active demethylation driven by enzymes of the TET family has been suggested [55].

DNMT3A and DNMT3B are tightly related in terms of amino acid sequence. Their interaction with histones is based on the Pro-Trp-Trp-Pro (PWWP) domain (centrally located) and on a zinc finger-binding domain that is called ATRX-DNMT3-DNMT3L (ADD). DNA methylation is implemented by the C-terminal catalytic domain. DNMT3B and DNMT3A are strongly expressed in embryonic stem cells that are undifferentiated, but their expression is low in adult somatic cells [81].

It was demonstrated not so long ago that the methylation of DNA and methylation of histones can synergistically regulate chromatin structure and function [93,94,95]. In particular, an analysis of a genome-wide DNA methylation pattern indicates that DNA methylation strongly correlates with H3K9me3 and H3K4me0 presence [96]. Of note, the colocalization of DNA methyl marks and histone marks is precisely governed by well-coordinated actions of “writers” and “readers” [97].

The ADD domain of DNA methyltransferases DNMT3A, DNMT3B, and DNMT3L recognizes the N-terminal end of histone H3. The DNA methylation activity of DNMT3A is switched on only when H3K4 is unmethylated [98,99] because the binding of the AAD domain to H3 is disrupted if its fourth lysine is methylated [97]. In turn, the unmethylated N-terminal tail of H3—judging by data obtained in vitro—allosterically stimulates the DNA-methylating activity of DNMT3A [100]. Thanks to a combination of biochemical and structural research projects, it has been found that DNMT3A exists in an autoinhibitory conformation. In this state, the interaction of the aforementioned catalytic domain with the AAD domain inhibits the binding of DNA in the catalytic site of DNMT3A. The binding of the AAD domain to the unmethylated N-terminal tail of H3 triggers a series of extensive conformational rearrangements within DNMT3A, which remove the autoinhibition and enable contact with DNA and methylation [101].

Of note, thymine DNA glycosylase (TDG) can interact with DNMT3A either through the PWWP domain or via the catalytic domain. Hypothetically, this interaction enhances the activity of TDG by promoting the binding of the enzyme to a site containing a mismatch. At the same time, the binding of TDG to DNMT3A suppresses the methyltransferase activity [102].

#### 2.2.2. The Role in the Organism

DNMT3A methylates specifically imprinted genes in germ cells and gene *Xist* on chromosome X; by contrast, DNMT3B can methylate satellite repeats in centromeres. In general, both methyltransferases are capable of methylating almost any DNA loci [103,104].

The different phenotypes of laboratory animals having a DNMT3A or DNMT3B knockout indicate that these enzymes, despite being highly similar, perform distinct functions in the organism. *Dnmt3a*^+/−^ mice and *Dnmt3b*^+/−^ mice develop normally and are fertile. *Dnmt3a*^−/−^ mice survive to birth, although they do not grow and die within 4 weeks afterward, likely because of a gastrointestinal dysfunction. A conditional *Dnmt3a* knockout in germ cells of males disturbs spermatogenesis and alters paternal imprints in differentially methylated regions in embryos. A conditional *Dnmt3a* knockout in germ cells of females induces changes in maternal imprints within differentially methylated regions, though paternal imprints are not affected. The *Dnmt3b* knockout is embryonically lethal because of developmental aberrations, e.g., growth failure and rostral defects in the neural tube. Nonetheless, a conditional *Dnmt3b* knockout in germ cells exerts no appreciable epigenetic or phenotypic effects [105]. A double knockout of *Dnmt3b* and *Dnmt3a* induces severe growth retardation and impaired morphogenesis after gastrulation [13]. At the molecular level, embryonic stem cells that are deficient in both *Dnmt3b* and *Dnmt3a* cannot initiate de novo methylation on pro-viral DNA and show the absence of de novo methylation throughout the genome, indicating the importance of DNMT3 for de novo methylation of DNA.

Lately, evidence suggests that DNA methyltransferases also take part in the formation of long-term memories. For instance, in memory event–activated hippocampal neurons, the closest early gene is expressed: *DNMT3A2*. DNMT3A2 localizes to more than a thousand promoters, methylates them, and suppresses their activity [106].

#### 2.2.3. SNPs, Deletions, and Suppression of Activity

At the moment, there are not many known diseases associated with mutations in *DNMT* genes in humans, which speaks volumes. The first mutations in a *DNMT* gene that were associated with a disease were mutations in DNMT3B, found in patients with immunodeficiency, centromere instability, and facial abnormalities (ICF) syndrome, which is named after the characteristic abnormalities observed during its development [13,46,47]. In a study on patients with ICF syndrome, Hansen and coworkers found three SNPs located in different regions of the *DNMT3B* gene, two of which create substitutions V726G and A603T, and one SNP that is located in an intronic part of the gene [47]. ICF syndrome is a recessive autosomal disease and features lowered levels of immunoglobulins in blood serum, and therefore most patients with this syndrome die of infectious diseases before reaching adulthood. Mild facial aberrations include low-set ears, epicanthal folds, hypertelorism, and macroglossia. Cytogenetic disturbances in lymphocytes are well pronounced: juxtacentromeric heterochromatin is highly elongated and filamentous in metaphase chromosomes, and this phenomenon is related to the formation of complex multiarm chromosomes [46].

Additionally, it has been found that intronic variant rs2424908 in *DNMT3B* correlates with an elevated risk of breast cancer [40]. Polymorphism C46359T in the *DNMT3B* promoter has been registered in patients with breast cancer [48]. Amplification of the *DNMT3B* gene is observed in breast cancer cells and has been implicated in resistance to DNA-demethylating therapeutics, e.g., 5-azacytidine (brand name Vidaza^®^), decitabine, and SGI-1027 [107].

Several research teams have determined that mutants of the DNMT3A enzyme are often present in patients with acute myeloid leukemia [41,42,108]. For example, amino acid substitution R882H in DNMT3A, often seen in patients having acute myeloid leukemia, appears to disturb DNMT3A function not only owing to a loss of enzymatic activity but also owing to the emergence of affinity between the active and inactive form of the protein, resulting in the formation of a catalytically inactive protein–protein conjugate [41,42,43,44,108]. Mutations in *DNMT3A* have also been found in tumor samples from several other hematological cancers [42,43,44,108].

Furthermore, 13 polymorphisms of the *DNMT3A* gene in Tatton–Brown–Rahman syndrome have been documented [45], which is a genetically determined syndrome causing tall stature, a distinctive facial appearance, and intellectual disability as well as the development of acute myeloid leukemia in approximately 25% of patients [41]. Furthermore, 11 of these substitutions are localized to functional domains of DNMT3A, and evidently, their emergence disturbs interaction between domains of the protein and binding to histones [45]. Ten of the SNPs result in a nonsynonymous amino acid residue substitution (I310N, G532S, M548K, C549R, L648P, P700L, R749C, N838D, F902S, or P904L), and one SNP in a deletion, Trp297del (in-frame deletion). Two more SNPs discovered by Tatton-Brown et al. in the *DNMT3A* gene cause a frameshift: Ser312fs or Arg767fs (frameshift deletion).

The fact that polymorphisms of DNMT3A and DNMT3B genes lead to various diseases once again confirms that functions in the organism differ between these enzymes.

DNMT3A mostly contributes to carcinogenesis, by regulating the expression of certain target genes. For instance, in one research article on this topic, it was shown that DNMT3A upregulation is associated with hypermethylation of promoters of genes *ERα* and *BRCA1* and accordingly with downregulation of estrogen receptor α (ERα) and of the product of breast cancer susceptibility gene 1 (BRCA1) [109]. On the other hand, stable suppression of the expression of oncoprotein SOX2 via DNMT3A overexpression slows down the development of a tumorigenic phenotype in breast cancer cells in mice [110].

### 2.3. DNMT2

Mammalian cells contain methyltransferase DNMT2 too, also known as TRDMT1. It was initially thought that it performs the DNA methylation function because this protein is a homolog of other members of this family. Nevertheless, it is now proven that DNMT2 takes part in cytosine-38 methylation in the anticodon loop of aspartate tRNA (this process is important for maintaining the stability of tRNA and methylation of RNA) and takes part in immune responses to many pathogens, e.g., RNA viruses [111,112,113].

## 3. Dioxygenases of the TET Family

In the past decade, much has been learned about the enzymology of demethylation of DNA. A big breakthrough in the understanding of this process was the discovery of enzymes of the TET family, which are capable of catalyzing the conversion of m^5^C into hm^5^C [114,115,116]. Modification hm^5^C appears to be the main intermediate in the pathway of demethylation of DNA bases. Currently, three members of this family are known: TET1, TET2, and TET3. As mentioned above, these proteins catalyze m^5^C oxidation to hm^5^C and further to f^5^C and ca^5^C. Next, ca^5^C and f^5^C and ca^5^C are removed from DNA by TDG, and as a consequence, an unmethylated nucleotide is restored by the functioning of BER enzymes [117,118,119,120,121].

**Figure 2:**
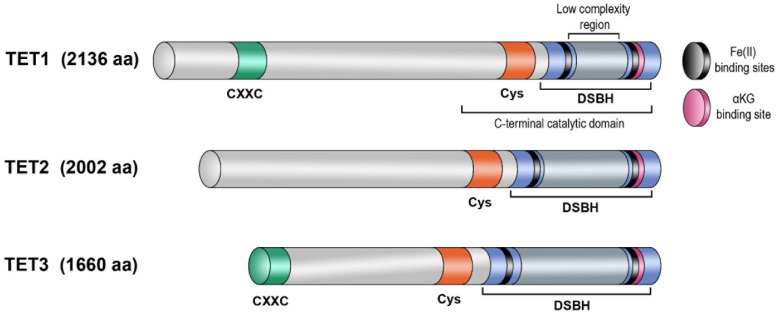
Domain structure of proteins of the TET family. The C-terminal catalytic domain, common among all members of the TET family, is composed of a cysteine-rich (Cys) domain, a DSBH domain, and binding sites for αKG and Fe(II).

The catalytic domain of enzymes of the TET family is situated within the C terminus of the protein and is composed of a double-stranded β-helical domain and a cysteine-rich domain (Figure 2) [7]. In the N-terminal part, TET3 and TET1 possess another domain, called CXXC, consisting of two Cys4-type zinc fingers. This domain mediates DNA binding [122]. The DSBH domain contains a long poorly structured region with an unknown function. TET1 and TET3 possess an N-terminal CXXC domain, which can directly associate with DNA and ensure the recruitment of the protein to target genomic sites [123].

The main function of mammalian TET proteins is thought to be ensuring the demethylation of epigenetic marks of DNA via the production of oxy-mC through a passive (replication-dependent) mechanism and an active (replication-independent) mechanism. The participation of a TET in the passive demethylation process is explained by the fact that DNMT1 is much less efficient at methylating position C5 of cytosine in CpG dinucleotides on newly replicated DNA strands if a product of oxidation of m^5^C (rather than m^5^C) is present on the template strand [124,125]. At the same time, more and more researchers claim that the role of the active mechanism (mediated by other proteins of BER) is much less than the role of the passive mechanism (connected to substrate specificity of DNMT1) [126].

Remarkably, the majority of eukaryotes that have one or more genes coding for homologous TET enzymes also have members of the DNMT family [127,128], possibly indicating a strong functional relation between these enzymes in eukaryotes.

Apparently, the work of a TET enzyme on DNA begins with transcription factors attracting these enzymes to enhancers from which m^5^C-mediated repression needs to be removed. This phenomenon plays a major role in the functioning of immune response cells because TET enzymes are reported to participate in the reactivation of immune-cell enhancers that govern a developmental switch within immature B lymphocytes and two signaling-dependent phenomena in mature B lymphocytes and regulatory T cells. The importance of TET enzymes in this case has been confirmed by the finding that inactivation of the genes encoding these enzymes quickly leads to malignant transformation of the cell [126].

To date, experimental data have been published about some polymorphic variants of TET enzymes associated with diseases (Table 2).

### 3.1. TET1

#### 3.1.1. Functional Characteristics

**Figure 3:**
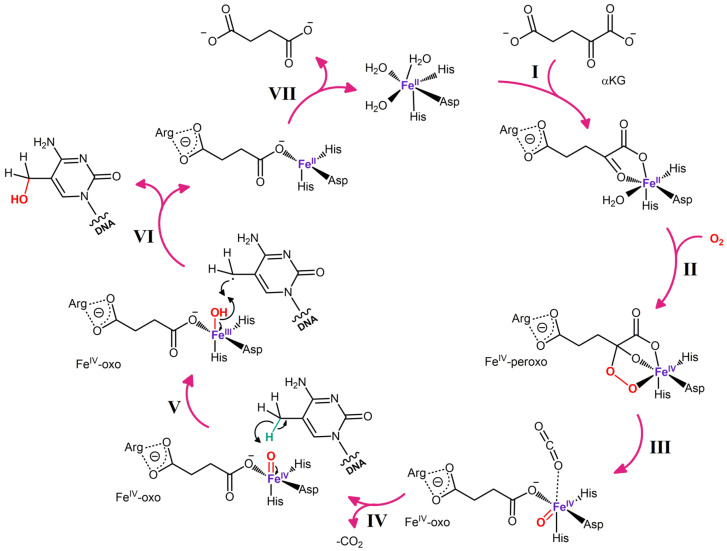
The proposed mechanism behind TET-driven oxidation of m^5^C. The demethylation reaction starts from the coordination of the Fe(II) and αKG to the conserved amino acid residues (His-Asp-His and Arg) of the catalytic center of the enzyme. In the second step, O_2_ displaces the water molecule from the Fe(II) coordination sphere. Then one of the bound oxygen atoms is transferred to the succinate molecule derived from αKG decarboxylation, and the other one is transferred to the Fe(II) to generate a high-valent Fe(IV)-oxo intermediate. The CO_2_ derived from the αKG decarboxylation leaves, and on the Vth step, the cleavage of the C-H bond of the m^5^C by the Fe(IV)-oxo oxidizing radical takes place, while the oxygen atom is transferred to the target carbon group through hydrogen abstraction. In the final step (VII) the coordination between restored Fe(II) and succinate molecule is disrupted. The same mechanism supposedly takes place in the case of hm^5^C and f^5^C. A radical rebound mechanism is depicted, but C–H activation may also proceed via a more concerted process.

TET1 was the first representative of the TET family to be identified and was discovered as a fusion partner of MLL in people with acute myeloid leukemia carrying translocation t(10;11) (q22; q23), whereas TET2 and TET3 have been subsequently identified based on their considerable homology with the TET1 sequence [131,132]. Later, it has been hypothesized that TETs belong to a larger superfamily: Fe(II)/αKG-dependent dioxygenases [128]. In the same year, it was found that they were indeed capable of oxidizing m^5^C [7], and soon, data were obtained about the mechanism of action of these enzymes (Figure 3). Just as other members of this superfamily, enzymes of the TET family use nonheme Fe(II) as a cofactor and αKG as a cosubstrate. To oxidize a substrate, these enzymes also require molecular oxygen, whereas CO_2_ and succinate are generated as byproducts of the reaction. Moreover, succinate, which is structurally similar to αKG, is an inhibitor of many representatives of the Fe(II)/αKG-dependent dioxygenase family; TET enzymes are no exception [133]. On the contrary, vitamin C, which apparently ensures the reduction of Fe(III) to Fe(II) within the enzyme’s active site, is an activator of such enzymes [134,135].

#### 3.1.2. The Role in the Organism

TET1 is linked with the removal of imprinting marks within germ cells that are primordial [136]. Research has revealed the crucial involvement of TET1 in physiological phenomena, e.g., development [136]. It has been shown that TET1 is expressed in the fetal brain, heart, and lung tissues; apparently, its expression in the adult heart, lungs, or brain stops or remains at undetectable levels. The expression of TET1 has also been found in adult skeletal muscle, thymus, and ovaries [131,132,137]. Two isoforms of TET1 have been identified in human cells. TET1 isoform 1 shows a homogenous nuclear pattern during the mitotic S phase, whereas isoform 2 is accumulated at sites of ongoing DNA replication in heterochromatin [138]. A deficit of TET1 has been shown to reduce the number of female germ cells by impairing the demethylation of meiosis-related genes [139], whereas a complete loss of TET1 is not critical for embryonic and postnatal development in mice [140]. TET1 is also important for the functions of intestinal stem cells in vivo [141] and is involved in complicated alterations of DNA methylation in the course of in vitro maturation of fetal intestinal epithelial organoids [142]. Epigenetic programming mediated by the catalytic activity of TET1 has been implicated in liver regeneration [143] and in remyelination within the mouse brain [144,145]. Mice deficient in TET1 exhibit impairments in spatial learning and memory [146]. Moreover, TET1 is required for fibroblast reprogramming for dopaminergic neurons [147]. Of note, TET1 overexpression leads to the upregulation of several memory-related genes but appears to impair contextual fear memory [148].

Evidently, the participation of TET1 in long-term-memory formation is related to its DNA-demethylating activity exclusively. When a hippocampal neuron is activated by a memory event, the topoisomerase 2β (TOP2B) dimer located on the promoter of a nearby early gene (*ERG1*) can cause a double-strand break within a region of the promoter. This double-strand break gets repaired after approximately 2 h but temporarily allows for the expression of the *ERG1* gene. ERG1 transfers TET1 to hundreds of promoters, thereby demethylating them and enhancing their expression [106].

#### 3.1.3. SNPs, Deletions, and Suppression of Activity

The role of TET1 in carcinogenesis remains controversial. The results of several studies have shown that in solid tumors, the amount of hm^5^C is reduced, and often, either protein expression of TET1 is diminished or this protein is completely excluded from the cell nucleus [149,150,151]. Conversely, another paper suggests that the *TET1* gene is a direct target of MLL (mixed leukemia) fusion proteins, and its expression is strongly enhanced during the progression of MLL-induced leukemia, thus inducing an overall increase in the amount of hm^5^C [152].

In the literature, there is currently almost no information on the association of polymorphisms in the *TET1* gene with human diseases. One of the few examples is a study by Pirola et al., which addresses the function of hm^5^C in DNA and of TET enzymes in nonalcoholic fatty liver disease pathogenesis [129]. They found that *TET1* polymorphism rs3998860 (producing substitution I1123M) significantly correlates with serum levels of cell death biomarker CK-18. Accordingly, cell cultures derived from the patient’s homozygous for this polymorphism proved to be more susceptible to apoptosis as compared to carriers of the normal allele. Allelic testing suggested that the TET1 variant I1123M is significantly associated with the severity of the disease in question [129].

### 3.2. TET2

#### 3.2.1. Functional Characteristics

It is worth mentioning that TET2 does not possess domain CXXC, thereby raising the question of how this enzyme binds to chromatin. One fairly sensible hypothesis is that the binding of TET2 to chromatin is mediated by the IDAX protein, which apparently has evolved from the CXXC domain of TET2 [153].

#### 3.2.2. The Role in the Organism

TET2 is broadly expressed throughout different human tissues, especially in hematopoietic cells [131,154]. Given that demethylation of DNA performs a crucial function in various biological processes, TET enzymes seem to be key to both physiological and pathological processes, as shown in many articles [123,155,156,157,158,159,160]. For instance, via modulation of DNA methylation, protein TET2 plays a crucial role in hematopoiesis [159]. The loss of TET2 activity leads to hypermutability in hematopoietic cell precursors, and this observation points to the key function of TET2 in the protection of cells from the accumulation of mutations [161].

#### 3.2.3. SNPs, Deletions, and Suppression of Activity

In various hematological cancers, one of the genes in which mutations are the most frequent is TET2 [162]. Mutations of the TET2 gene are detected as often in lymphomas, especially in their T-cell types [163,164]. Nonetheless, data on how much the overall survival of patients with acute myeloid leukemia correlates with TET2 mutations have been inconsistent, and hence no clear conclusion can be drawn [58].

A project aimed at demonstrating the benefits of next-generation sequencing in clinical mutation profiling has also revealed that the *TET2* gene contains mutations in 28.7% of tested patients having myelodysplastic syndromes or myelodysplastic–myeloproliferative cancers, with some simultaneously carrying two or three pathogenic mutations in this gene [165]. Missense mutations in this context are concentrated mainly in the C-terminal catalytic domain [166]. Substitutions in TET2 that are associated with myeloid tumors have been shown to diminish the enzymatic activity and as a consequence decreased amounts of hm^5^C in genomic DNA [167]. In addition, under-expression of TET2 is observed in glioblastoma and other gliomas compared to normal human brain tissue [168,169].

Even in the absence of mutations in the coding region of *TET* genes, a loss of TET function and low levels of hm^5^C are often seen in many types of cancers [167,170,171,172,173], including hematological cancers and solid tumors. This phenomenon may be mediated by zero expression or inactivation of functions of TET proteins at various stages of gene expression, e.g., transcriptional suppression via hypermethylation of the promoter of a *TET* gene, post-transcriptional processes such as microRNA-driven silencing, and accelerated degradation; the reason is that post-translational modifications have different effects on the stability of TET proteins [174,175,176,177]. Additionally, hypoxia and metabolic changes can lead to a loss of TET function owing to disturbances in the enzymatic activity of these proteins (and of other dioxygenases).

Most studies on TET2 mutation-associated cancers have been centered around the observation that a loss of function in TET proteins causes enhanced methylation of the genomic regions where such proteins play transcription-regulatory roles. This local DNA hypermethylation, which takes place mostly in enhancers and promoters, can cause transcriptional repression of tumor suppressor genes and of genes taking part in DNA damage repair, thereby promoting tumorigenesis [178,179]. Indeed, focal hypermethylation of DNA is usually observed in tumors featuring deficient activity or expression of TET proteins.

### 3.3. TET3

#### 3.3.1. Functional Characteristics and the Role in the Organism

Gu et al. have reported that TET3 is possibly required for the removal of parental (mainly paternal) methyl marks in the zygote [180]. It was shown in their work that the mouse TET3 homolog is localized to the male and female pronucleus during the zygotic stage, and its localization shifts to the cytoplasm at other preimplantation stages [180]. As a member of the TET family, TET3 plays an important part in demethylation within many biological phenomena such as the formation of a zygote [180,181,182,183], embryogenesis [184], regeneration of axons [185], and synaptic transmission [186]. Similarly to TET1, TET3 is expressed in the fetal brain but not the adult brain [131]. TET3-mediated demethylation of DNA is required for liver tissue maturation because this demethylation ensures proper expression of hepatocyte genes [187].

Not so long ago, TET3 was shown to perform a function in the reduction of synaptic strength in hippocampal neurons [186]. Of note, in contrast to hippocampus neurons, TET3—not TET1—is expressed in an activity-dependent manner inside cortical neurons [188]. Gephyrin stabilizes GABA receptors located on the postsynaptic membrane and participates in the extinction of fear [189]. Furthermore, Li et al. have confirmed an elevated amount of TET3 at the gephyrin locus, where they have demonstrated increased occupancy of TET3 together with accumulation of an intermediate demethylation mark (hm^5^C) [188].

#### 3.3.2. SNPs, Deletions, and Suppression of Activity

Weakened catalytic activity of TET3 is related to intellectual disability and abnormal growth, implying a crucial function of TET3 in development [130]. Loss of TET3 function has turned out to be a common feature among 11 patients showing signs consistent with a Mendelian disorder resulting from disturbances of epigenetic regulatory mechanisms. The cause was determined in that work: the emergence of one or several missense variants in the strongly conserved catalytic domain within TET3, most of which were functionally confirmed to diminish the activity of TET3 (substitutions R752C/V1089M, V908L, F1072C/A1076T, T851M, and P1677L), to generate a frameshift (H1660Pfs*52 or W406Gfs*135), or to create a nonsense variant (Gln1695*).

In adult mice, TET3 inactivation is linked with anxiety-like behavior, although the molecular mechanisms underlying this connection are yet to be investigated [190].

Furthermore, there is under-expression of TET3 in glioma, and this observation is explained by a genome-wide decline of hm^5^C amounts as compared to the normal brain [191]. Moreover, downregulation of TET3 correlates with a worse prognosis of progression of this disease [191].

## 4. Conclusions

Using enzymes of two families—DNMT (DNA methyltransferases) and TET (DNA dioxygenases)—as an example, this review provides up-to-date information about enzymes responsible for epigenetic methylation or demethylation of DNA and for the maintenance of a methylome state in the human genome. The totality of the published data is suggestive of a relation between the level of expression/activity of these enzymes and the development of various pathologies. In addition, the effects of natural SNPs in genes of these enzymes on the efficiency of their activity are examined here, and possible consequences for carriers of such substitutions are described. It should be noted that cell lines and/or transgenic animals carrying functionally defective genes of these enzymes can serve as helpful tools for determining the biological roles of these proteins in vivo and for confirming the relevance of their functions to the development of certain diseases. In the future, major breakthroughs can be expected in the field of research on organisms with double knockouts of both methylation genes (*DNMT*) and genes of active demethylation (including *TET* genes and genes of BER enzymes). These accomplishments will help to identify possible reserve systems that participate in the regulation and coordination of interactions of these pathways, thereby ultimately ensuring a certain methylation state of genomic DNA. Moreover, investigation into these enzymatic cascades will facilitate the search for new targets for directed modulation of the activity of enzymes that ensure the balance between processes of DNA methylation and demethylation.

## Figures and Tables

**Table 1:** DNMT genes’ SNPs associated with various disorders and diseases.

Gene	Associated Diseases/Disorders	Amino Acid Substitution in Protein	Location of Mutations	References
*DNMT1*	Hereditary sensory autonomic neuropathy type 1E (HSAN1E)	C353F, T481P, D490E/P491Y, P491L, Y495H, Y495C, P507N, K521del, Y524D, I531N, H569R	RFTS domain	[34,35,36,37]
	Autosomal dominant cerebellar ataxia, deafness and narcolepsy (ADCA-DN)	A570V, V606F, G605A, C596R	RFTS domain	[36,38,39]
	High risk of breast cancer	H97R	DMAP1 domain	[40]
*DNMT3A*	Acute myeloid leukemia	R882H, R882C	MTase catalytic domain	[41,42,43,44]
	An overgrowth syndrome with intellectual disability (Tatton–Brown–Rahman syndrome)	W297del (c.889_891delTGG)	PWWP domain	[45]
		G532S, M548K, C549R	ADD domain	
		L648P, P700L, R749C, N838D, F902S, P904L, Arg767fs	MTase catalytic domain	
*DNMT3B*	Immunodeficiency, centromere instability, and facial abnormalities (ICF) syndrome	A603T, V726G, D809G	MTase catalytic domain	[46,47]
	High risk of breast cancer	rs2424908	Intronic variant	[40]
	Breast cancer	C46359T	Promoter area	[48]

Del denotes an in-frame trinucleotide deletion leading to the loss of the single amino acid; fs means a frameshift insertion or deletion of a single nucleotide at the codon of the corresponding amino acid. MTase: methyltransferase.

**Table 2:** TET genes’ SNPs associated with various disorders and diseases.

Gene	Associated Diseases/Disorders	Amino Acid Substitution in Protein	Location of Mutation	References
*TET1*	Nonalcoholic fatty liver disease	I1123M	Disordered region	[129]
*TET2*	Type 2 diabetes	I1762V	DSBH domain	
*TET3*	Mendelian disorder	T851M, V908L	Cis-rich domain	[130]
		R752C/V1089M, F1072C/A1076T, P1677L, H1660Pfs (c.4977_4983del), Gln1695* (nonsense variant)	DSBH domain	
		W406Gfs (c.1215delA)	-	

Fs denotes a frameshift deletion of several nucleotides starting from the codon of the corresponding amino acid. * Stands for the nonsense mutation.

## Data Availability

Data are available upon request to A.T.D. Tel.: +7-383-363-5174, E-mail: davleta94@gmail.com.
